# Breast Implant Illness: A Cohort Study

**DOI:** 10.7759/cureus.38056

**Published:** 2023-04-24

**Authors:** Thomas J Serena, Peter Habib, Amy Derosa

**Affiliations:** 1 General Surgery, Beaumont Health, Livonia, USA; 2 General Surgery, Beaumont Health, Farmington Hills, USA; 3 Plastic and Reconstructive Surgery, Beaumont Health, Farmington Hills, USA

**Keywords:** breast implant complications, capsulectomy, breast implants, breast augmentation, breast implant illness

## Abstract

Background

Breast implant illness (BII) is a clinical disease defined by a constellation of symptoms that patients experience as a result of their breast implants. This retrospective, cohort study evaluated the benefit of breast implant explantation with total capsulectomy on patients’ symptoms.

Methodology

This is a single-center, single-arm, cohort study utilizing retrospectively collected data. All participants included in this study voluntarily presented to the department of plastic and reconstructive surgery and requested breast implant removal. A total of 229 patients were enrolled in the study over a three-year period from 2018 to 2021. The primary endpoints of the study were to objectively grade the improvement of symptomatology following surgical intervention. The secondary endpoints were to identify co-factors such as age, comorbidities, implant characteristics, the timing of symptoms, and other data that were potentially influenced by or influencers of the breast implant illness.

Results

The study achieved a total of 549-point decrease in symptom frequencies following surgery. Furthermore, with an average preoperative symptom score of 3.5 (scored 1-5) and a postoperative average of 1.9, the study demonstrated a score reduction of 1.6 across all symptoms. Furthermore, the study was able to eliminate on average 2.8 symptoms of breast implant illness from every patient following explantation.

Conclusion

Breast implant illness is a true clinical entity that affects an extensive population of patients who have undergone breast augmentation. This study has not only highlighted the extensive morbidity of breast implant illness but has also demonstrated that there is an opportunity to standardize treatment for this disease. These outcomes have proven that a significant reduction in disease severity can be achieved with breast implant explantation and total capsulectomy.

## Introduction

The history of breast augmentation has grown from trial and error over the course of the last 60 years. The first silicone implants were placed in 1962 by Cronin and Gerow [1]. Over four million women across the globe have undergone augmentation for a multitude of reasons including but not limited to oncological reconstruction, cosmesis, or trauma. It is evident that this procedure does not come without risks as over 400 reports have been filed regarding symptoms and health conditions believed to be associated with implants [2]. The United States Food and Drug Administration has drawn attention to the controversies of breast augmentation and its safety. While large studies have not identified an association between breast implants and connective tissue disorders, there have been links demonstrated between implants and lymphoma, autoimmune diseases, and physical deformities [3-6]. Recently, a new disease process has manifested itself, known as breast implant illness (BII).

Breast implant illness is a clinical disease defined by a constellation of symptoms patients experience as a result of their breast implants. As the disease has become more popular in the media, widespread conversations have characterized this illness as a cluster of over 50 symptoms associated with breast implants [7]. It is unknown whether or not this is secondary to a direct pathological process or perceived connection to implant placement. Patients suffering from BII most commonly present with complaints of fatigue, anxiety, chronic pain, and exacerbations of endocrine, autonomic, and peripheral nervous system dysfunctions. In select groups, patients will even manifest somatic dysfunctions.

Comprehensive studies have been conducted regarding foreign body reactions within the body. The first description of patients’ reaction to breast implants was described in 1960 and called human adjuvant disease [8]. More recently, a condition known as autoimmune/autoinflammatory syndrome induced by adjuvants (ASIA) has highlighted a body’s propensity to pathologically react to a variety of foreign materials. Adjuvants are agents that carry the capability to induce immune reactions and have been added to vaccines to increase patients’ immunologic response to vaccination [6,9]. Silicone has been considered largely an inert material and for that reason has been used in medical devices. However, there is evidence to suggest that silicone exposure is related to the development of autoimmune diseases and autoantibodies in patients following silicone implants [10].

As unexplained symptoms have continued to manifest in women with breast implants, there has been increased pressure to identify if this is a matter of correlation or causation. A descriptive cohort study was performed in Amsterdam that studied 80 women with symptoms believed to be related to their breast implants. After excluding alternative explanations, a clear pattern of signs and symptoms was recognized. Most women had a history of allergies, suggesting that intolerance to silicone or other substances in the implants might cause their symptoms. In 69% of women, the explantation of implants reduced or eliminated symptoms [11].

In an alternative study, researchers compared 100 patients with ASIA due to “silicone implant incompatibility syndrome” diagnosed in 2014 in Maastricht, Netherlands, with 100 historical patients with “adjuvant breast disease” diagnosed in the Baylor College of Medicine, Houston, USA, between 1985 and 1992. Of the 54 patients who underwent removal of their silicone breast implant, 50% (n=27) of the patients experienced an improvement in complaints after the explantation of the implant [12]. Other studies from the Netherlands have demonstrated up to 69% of women with explantation of implants having complete symptom resolution [11].

A standard of care for breast implant illness does not currently exist. This is owed to the paucity of literature on the topic. The presentation and management of BII have been based largely on anecdotal evidence. The aim of this study is to remedy this by providing a summary of our current knowledge of the illness, propose a standardized approach to management, and qualify and quantify the outcomes of an innovative treatment. We have previously identified a small cohort of patients who successfully underwent breast implant explantation with total capsulectomy with significant resolution of their symptoms [13]. This cohort study evaluated the efficacy of breast implant removal with total capsulectomy on patients’ symptoms.

## Materials and methods

**Figure 1:**
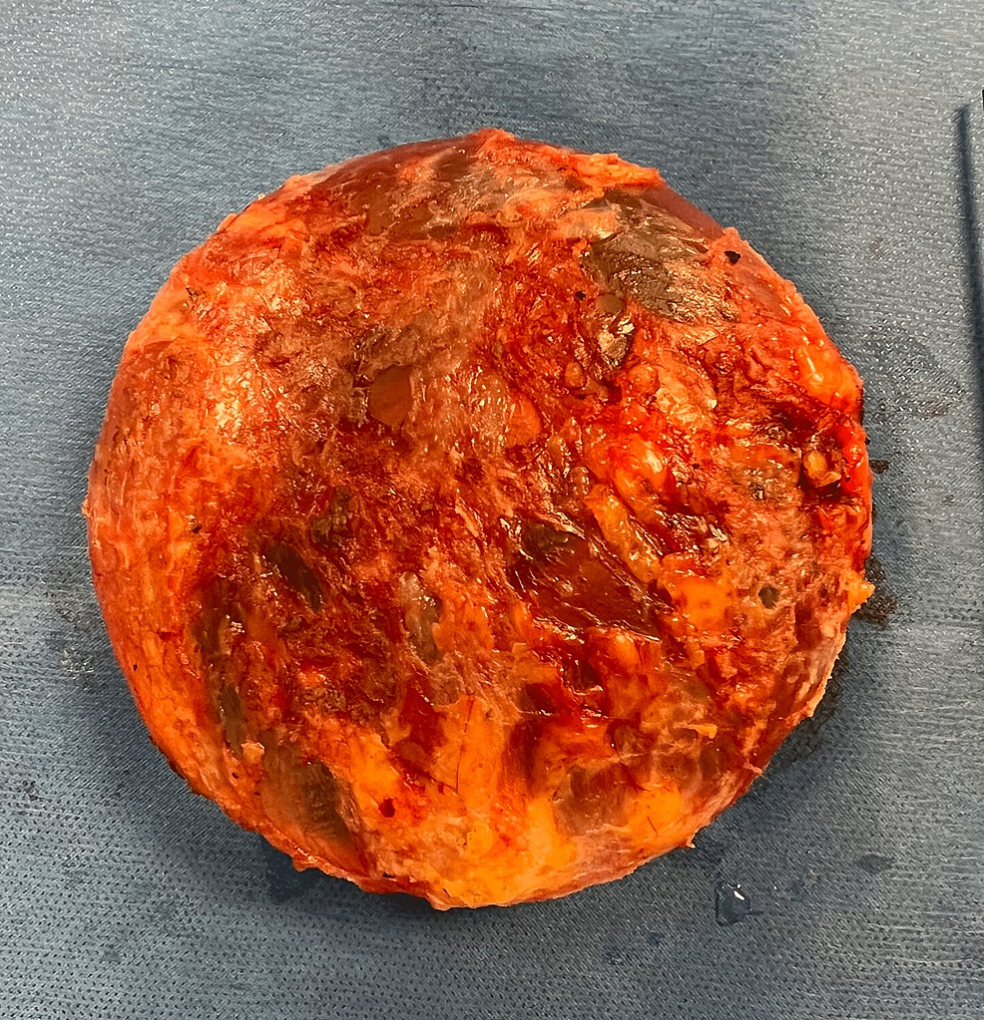
Postoperative image demonstrating completed breast implant explantation and total capsulectomy. The procedure performed is as follows. The surgical incision is to encompass previous scars or facilitate dissection. Dissection is carried through the subcutaneous tissue until the implant capsule is encountered. The capsule and implant are carefully excised with sharp dissection and removed from the breast. The breast cavity is copiously irrigated with sterile saline and hemostasis is achieved with electrocautery. A JP drain was placed within the pocket and sutured to the chest wall as it exited laterally with a nylon stitch. The deep tissue and muscle were approximated with layers of 2-0 absorbable sutures. Skin edges were approximated with 4-0 absorbable monofilament followed by a running 4-0 absorbable monofilament. JP drain: Jackson-Pratt drain

This is a single-center, single-arm, cohort study. All participants included in this study voluntarily presented to the plastic and reconstructive surgery service and requested breast implant removal. All patients requesting surgery were offered participation in the study and were then screened for eligibility. The study’s inclusion criteria were patients requesting removal of breast implants, nonpregnant patients, age greater than or equal to 18 years old, and patients with the ability to complete a survey. Patients falling outside these parameters were excluded. Patients included in the study underwent breast implant explantation with total capsulectomy defined as the removal of the breast implant and the entirety of the surrounding inflammatory capsule from the patient’s body. The standardized procedure is presented in Figure 1 with a photograph of the completed total capsulectomy.

**Figure 2:**
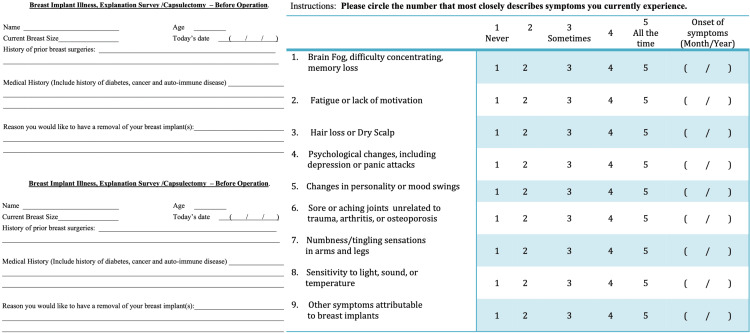
Preoperative and postoperative surveys completed by patients.

All patients completed a standardized preoperative and postoperative survey at two months ± two weeks after the operation, as seen in Figure 2. Patients’ descriptive data were summarized as counts and percentages for categorical data (patient-reported symptoms) and means with corresponding standard deviations for numerical data (age, duration of symptoms, etc.). Patients participating in the study came from multiple states across the nation, and given widespread travel, a population of patients underwent computer-assisted telephone interviews (CATI).

The primary endpoints of the study were to objectively grade the resolution of symptomatology following surgical intervention. The secondary endpoints were to identify co-factors such as age, comorbidities, implant characteristics, the timing of symptoms, and other data, which were potentially influenced by or influencers of the patient’s illness. In addition to standard analysis, a subgroup analysis was performed to assess extremes of patient outcomes with the greatest and least improvement.

Statistical analysis was performed for the primary study objective revealing that a sample of 199 patients would need to be included in the study to show a 1% margin of error at a 95% confidence level. A further prediction of pre-/post-differences and cross-comparisons of nonparametric data was performed using the conventional power level of 80% and a two-tailed alpha of 0.05 and assuming a medium effect size. This demonstrated that if 114 completed patient surveys, it would achieve a 1% margin of sampling error. Given this is a relatively understudied illness, any sample size greater than 196 patients would best serve to produce a representative description of a true patient population.

## Results

A total of 229 patients were enrolled in the study over a three-year period from 2018 to 2021. Thirty patients were excluded secondary to 25 patients having failed to follow up and five patients with failure to complete post-surgical forms. For the totality of our analysis, 199 patients were assessed, of which 100% were female. Patient demographics and specifics regarding surgery did not vary significantly. The mean age of breast implant placement was 34.58 years. The mean age of breast implant removal was 47.44. Patients possessed implants for an average of 12.86 years. There were 53 patients who were unable to provide accurate dates for the age at implantation.

The reported data showed that 95 patients had saline implants and 99 silicone. Textured implants were removed in 53 patients, and non-textured implants were removed in 141 patients. Rupture was present in 22 patients, and intact implants were present in 171 patients. Across saline versus silicone, textured versus non-textured, and rupture versus non-rupture, the patients did not display significant differences between the categories. Preoperative and postoperative breast sizes were scored. This subjective data was transferred onto an ordinal system with numbers assigned across sizes. A significant portion of patients (81) failed to submit this data.

**Figure 3:**
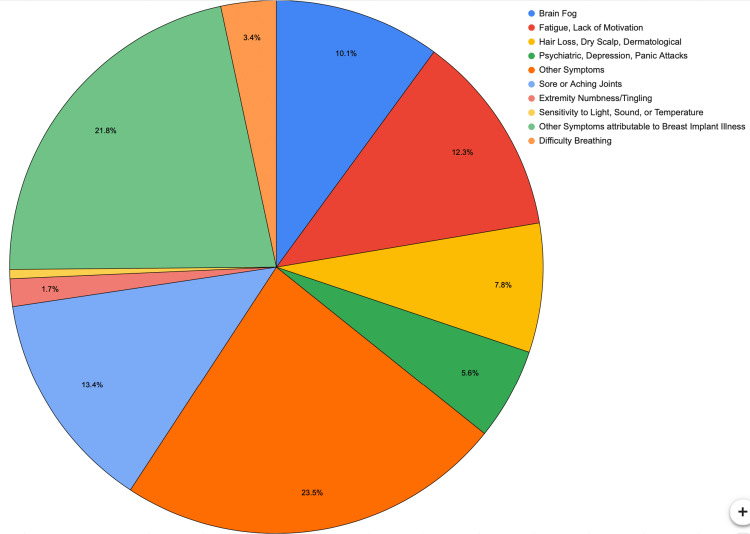
Primary symptoms that patients reported as reasons to undergo breast implant explantation. This is a visual representation of the most commonly reported symptoms.

Patients were asked to write down the primary symptoms for which they were seeking implant removal. Of the patients who responded, the largest categories were 42 (21.1%) patients with chest discomfort, 22 patients (11%) who reported fatigue and lack of motivation, and 24 (12.2%) patients who reported aching or sore joints as primary symptoms. There were also 39 (19.%5) patients who reported other symptoms as a main driving force for the removal of implants. The results can be conceptualized in Figure 3.

Past medical history was also recorded among patients. The patient population has an extensive past medical history with the top three categories being endocrine disease (n=37), autoimmune disease (n=35), and anxiety/depression/psychiatric disease (n=27). Other common conditions reported in our patient population include cancer, gastrointestinal disease, and cardiopulmonary disorders.

The frequency at which patients experienced the symptoms of the nine domains was obtained in a survey, which included the following: brain fog, fatigue/lack of motivation, hair loss/dry scalp/dermatological conditions, psychiatric disease/depression/panic attacks, personality changes/mood swings, sore/aching joints, numbness/tingling in arms or legs, sensitivity to light/sound/temperature, and other symptoms attributable to breast implant illness. These domains were scored as depicted in the survey and represented in Table 1 with scores of 1 meaning never having symptoms, 3 meaning sometimes, and 5 meaning having symptoms all the time. The average results are shown in Table 2.

**Table 1:** Before and after surgery comparisons of the number of patients reporting different levels of symptom frequency. Objective scores were reported in the survey with a score of 1 equating to never having symptoms, 3 representing sometimes having symptoms, and 5 meaning having symptoms all the time.

N=199	Objective score	Number of patients in score category	Percentile	Number of patients in score category	Percentile
		Before removal		After removal	
Brain fog	5	85	42.7%	6	3%
	4	64	32.2%	11	5.5%
	3	43	21.6%	55	27.6%
	2	3	1.5%	69	34.7%
	1	4	2%	58	29.1%
Fatigue and lack of motivation	5	92	46.2%	4	2%
	4	73	36.7%	13	6.5%
	3	29	14.6%	50	25.1%
	2	4	2%	74	37.2%
	1	1	0.5%	58	29.1%
Hair loss, dry scalp, and dermatological condition	5	76	38.2%	11	5.5%
	4	37	18.6%	22	11.1%
	3	33	16.6%	25	12.6%
	2	24	12.1%	46	23.1%
	1	30	15.1%	85	42.7%
Psychiatric disease, depression, and panic attacks	5	48	24.1%	3	1.5%
	4	55	27.6%	5	2.5%
	3	51	25.6%	42	21.1%
	2	25	12.6%	55	27.6%
	1	20	10.1%	94	47.2%
Personality changes and mood swings	5	37	18.6%	0	0%
	4	58	29.1%	3	1.5%
	3	56	28.1%	29	14.6%
	2	27	13.6%	76	38.2%
	1	21	10.6%	91	45.7%
Sore or aching joints	5	106	53.3%	9	4.5%
	4	43	21.6%	18	9%
	3	25	12.6%	31	15.6%
	2	12	6%	69	34.7%
	1	13	6.5%	72	36.2%
Numbness or tingling in arms or legs	5	42	21.1%	2	1%
	4	53	26.6%	16	8%
	3	55	27.6%	25	12.6%
	2	21	10.6%	45	22.6%
	1	28	14.1%	111	55.8%
Sensitivity to light, sound, or temperature	5	71	35.7%	5	2.5%
	4	32	16.1%	15	7.5%
	3	41	20.6%	32	16.1%
	2	23	11.6%	42	21.1%
	1	32	16.1%	105	52.8%
Other symptoms attributable to breast implant illness	5	37	18.6%	5	2.5%
	4	4	2%	4	2%
	3	18	9%	25	12.6%
	2	45	22.6%	45	22.6%
	1	95	47.7%	120	60.3%

**Table 2:** Average preoperative and postoperative scores with average score change, across all symptom domains, before versus after surgery.

N=199	Average preoperative score	Average postoperative score	Average difference
Brain fog	4.1	2.2	1.9
Fatigue and lack of motivation	4.3	2.2	2.1
Hair loss, dry scalp, and dermatological condition	3.5	2.0	1.6
Psychiatric disease, depression, and panic attacks	3.4	1.8	1.6
Personality changes and mood swings	3.3	1.7	1.6
Sore or aching joints	4.1	2.1	2.0
Numbness or tingling in arms or legs	3.3	1.8	1.5
Sensitivity to light, sound, or temperature	3.4	1.9	1.6
Other symptoms attributable to breast implant illness	2.2	1.6	0.6
Average	3.5	1.9	1.6

The symptoms with the highest pathological score reported by patients were fatigue or lack of motivation, brain fog, and sore or aching joints with average scores of 4.3, 4.1, and 4.1, respectively, on preoperative surveys. These scores decreased to 2.2, 2.2, and 2.1, respectively, for an average two-level decrease in symptoms. Of note, the average score for all patients in the study for each and every category besides other symptoms attributed to breast implant illness was greater than 3 correlating with patients regularly experiencing these symptoms in their life. The average total score for all preoperative symptoms was 3.5 with a postoperative average of 1.9 for a total score reduction of 1.6. In addition, the top three symptom domains experienced the greatest reduction in scores based on postoperative surveys. Strikingly, in comparison of preoperative and postoperative scores, 550 scores decreased to a score of 1 consistent with never experiencing symptoms again following breast implant explantation. This is an average of 2.8 symptoms per patient, which they never experienced again. Furthermore, when assessing patients who preoperatively scored a 5, meaning they experienced their symptoms all the time, 549 scores decreased in any magnitude from a 5 to a lower frequency. This again is an average of 2.8 symptoms decreasing to no longer affecting each and every moment of the patients’ lives.

We then performed a subgroup analysis of patients scoring the highest score differences between pre-surgical and post-surgical surveys. Scores were reported across eight symptom classes plus an additional category defined as other symptoms attributable to breast implant illness. Across the nine total domains, the maximum score for symptom severity was 5 and the lowest score for symptom severity was 1. The greatest improvement possible across surveys was 36 points, as calculated by nine categories with a maximum improvement of 4.

We created subgroup A and subgroup B, referring to the 20% of patients with the greatest improvement and the 20% of patients with the least improvement in their change of symptomatology prior to and following surgery. Subgroup A had implants for a shorter period of time than subgroup B, with an average of 10.1 versus 17.2 years. When cross-analyzing score improvement across rupture, 23.9% of patients with intact implants scored into subgroup A, while only 9% of patients with ruptured implants scored into subgroup B. Subgroup A also contained the highest proportion of patients suffering from an autoimmune disease, endocrine disease, psychiatric disease, and pain/discomfort. Patients reporting a history of autoimmune disease had 34.2% placement into subgroup A, demonstrating a strong propensity to benefit the greatest from implant removal. The remaining categories with a strong propensity for improvement were patients experiencing pain and discomfort (37.5%), psychiatric disease (25.6%), and endocrine disease (24.3%).

## Discussion

The current study demonstrates that breast implant illness is more than an anecdotal collection of symptoms. The tested theory was validated in that not only did the patients quantify a significant level of disease, but they also exhibited a powerful reduction in symptomatology following surgery. The study achieved a total of 549 score decrease in frequencies of symptoms following surgery pointing to a quantifiable decrease in morbidity. Furthermore, with an average preoperative symptom score of 3.5 and a postoperative average of 1.9, the study demonstrated a score reduction of 1.6 across all symptoms. Furthermore, the study was able to eliminate on average 2.8 symptoms of breast implant illness from every patient following explantation. These numbers alone serve to highlight the tremendous improvement in morbidity the patients experienced. Most importantly, this highlights the importance of developing a standardized approach to treatment for patients suffering from breast implant illness. Given that patients’ postoperative surveys were completed two months following surgery, future studies could follow the long-term outcomes of patients to evaluate the durability of surgical intervention.

The subgroup analysis was peculiar in that patients with the top 20% greatest improvement in their symptoms had implants for an average of 7.1 years less than patients in the lowest 20% of improvement. This may point to the prolonged inflammatory response being more difficult to clear after patients have implants for an extended period of time. However, it may be a reflection that patients with less severe symptoms had delayed presentation. Further important characteristics of patients with the greatest improvement in their symptoms following the explanation included a 14.9% increase in rupture of implants between the top and lowest improvers following explanation. While this number itself is not as high as anticipated, it suggests a higher improvement for patients who ruptured their implants.

All patients included in the study underwent breast implant explantation with total capsulectomy. We believe total capsulectomy to be the key to success for the patient population. One theory may relate to improving lymphatic drainage secondary to the complete excision of a foreign body. However, it is our belief that the illness is overall caused by the systemic inflammatory reaction caused by both the implants and the inflammatory capsule. This is the reason we chose to perform a complete capsulectomy as opposed to leaving the implant capsules in vivo. Further studies could objectively quantify this data by following trends in inflammatory markers before and after surgical intervention.

There are several limitations of the study. First, it should be noted that the patients included in the study seek to have explanations because they believe they have breast implant illnesses. For this reason, there may be a degree of self-fulfilling prophecy as the patients believe that their symptoms will resolve following surgery. This could be further backed by some symptoms of this disease being related to anxiety or depression related to the patients’ experience with their implants. While this is difficult to ignore, it is important to highlight that the greatest improvement following the explanation relates to patients with endocrine and autoimmune-related symptoms. This serves to highlight that the psychological component of the illness is not as prominent as patients with the greatest improvement following surgery.

Further limitations of the study are inherent to its retrospective cohort nature. While the study had adequate power, there were a significant proportion of patients who dropped out of the study. Furthermore, there were 53 patients who failed to provide the totality of information requested on the survey and five patients who failed to follow up or complete their surveys. This may be explained in part by the study occurring in the middle of the COVID-19 pandemic that significantly altered standard patient care and follow-up. With that said, we believe that the patients in the study captured a strong representation of patients suffering from breast implant illness.

## Conclusions

Breast implant illness is a true clinical entity that afflicts an extensive patient base, who have undergone breast augmentation. This study has not only highlighted the extensive morbidity of breast implant illness but has also demonstrated that there is an opportunity to standardize treatment for this disease. The results have demonstrated that a significant reduction in disease severity can be achieved with breast implant explantation and total capsulectomy. Furthermore, it has highlighted that patients with autoimmune disease, endocrine disease, and anxiety/depression/psychiatric disease have a predisposition to more significant disease severity. We hope that the data draws attention to this disease, for both patients and practitioners, to standardize treatment for breast implant illness.
